# Consistency of systematic chemical identifiers within and between small-molecule databases

**DOI:** 10.1186/1758-2946-4-35

**Published:** 2012-12-13

**Authors:** Saber A Akhondi, Jan A Kors, Sorel Muresan

**Affiliations:** 1 Department of Medical Informatics, Erasmus University Medical Center, P.O. Box 2040, Rotterdam, CA, 3000, Netherlands; 2 Chemistry Innovation Centre, Discovery Sciences, AstraZeneca R&D Mölndal, Mölndal, S-431 83, Sweden

**Keywords:** Molecular structure, Chemical databases, Systematic chemical identifiers, Quality control, InChI, SMILES, IUPAC

## Abstract

**Background:**

Correctness of structures and associated metadata within public and commercial chemical databases greatly impacts drug discovery research activities such as quantitative structure–property relationships modelling and compound novelty checking. MOL files, SMILES notations, IUPAC names, and InChI strings are ubiquitous file formats and systematic identifiers for chemical structures. While interchangeable for many cheminformatics purposes there have been no studies on the inconsistency of these structure identifiers due to various approaches for data integration, including the use of different software and different rules for structure standardisation. We have investigated the consistency of systematic identifiers of small molecules within and between some of the commonly used chemical resources, with and without structure standardisation.

**Results:**

The consistency between systematic chemical identifiers and their corresponding MOL representation varies greatly between data sources (37.2%-98.5%). We observed the lowest overall consistency for MOL-IUPAC names. Disregarding stereochemistry increases the consistency (84.8% to 99.9%). A wide variation in consistency also exists between MOL representations of compounds linked via cross-references (25.8% to 93.7%). Removing stereochemistry improved the consistency (47.6% to 95.6%).

**Conclusions:**

We have shown that considerable inconsistency exists in structural representation and systematic chemical identifiers within and between databases. This can have a great influence especially when merging data and if systematic identifiers are used as a key index for structure integration or cross-querying several databases. Regenerating systematic identifiers starting from their MOL representation and applying well-defined and documented chemistry standardisation rules to all compounds prior to creating them can dramatically increase internal consistency.

## Background

The past decade has seen a major increase in the availability of public and commercial chemical databases [1]. Resources such as PubChem (released in 2004) [2] and ChEMBL (released in 2009) [3], with their corresponding web services, have gained the trust of many researchers in the fields of cheminformatics, bioinformatics, systems biology, and translational medicine. Because large numbers of compounds and associated structure-activity relationships (SAR) data are published in journals and patents every year, many new data sources have become available, each covering different aspects of the connectivity between the SAR-related entities [4]. With the increasing usage of these resources by scientists from both academia and the pharmaceutical industry, quality control of chemical structures and associated metadata is becoming a necessity [5].

Correctness of a structure extracted from databases has a great impact on predictive ability of computational models for quantitative structure-activity relationships (QSAR) [6]. A recent study by Williams and Ekins [7] on a subset of a chemistry database showed more than 70% errors in the absolute structural integrity, a striking difference to the 5-10% level the authors had anticipated. In another study of database quality, Oprea et al. [8] have illustrated how errors within a database are transferred to other databases following data integration (also mentioned by Williams et al. [9]). Quality issues have also been observed in the relationship between chemical structures and the corresponding identifiers, such as chemical names referring to structures with different stereochemistry or CAS numbers incorrectly associated with a particular salt or mixture [9]. Although these problems are known to exist, there have been no studies that quantify the consistency between structures and their identifiers.

Chemical identifiers can be distinguished in two major classes based on how they are generated. The first consists of systematic identifiers, which are generated algorithmically and should have a one-to-one correspondence with the structure (however, different software could generate different flavours, as is the case for SMILES notations [10,11]). The second class comprises non-systematic chemical identifiers. These are source dependent and usually generated at the point of registration within a particular source (e.g. CAS numbers, PubChem compound identifiers (CIDs) and substance identifiers (SIDs), generic or drug brand names).

Structure depictions are the natural language for chemists. In order to convert the images to a form usable by computers, several file formats and chemical identifiers have been introduced. The MOL file format [12], SMILES notations [10], InChI strings [13], and IUPAC names [14] are arguably the most widely used. In the context of this work we will refer to IUPAC names, SMILES notations, and InChI strings as systematic identifiers.

Most chemical databases are built starting from the MOL file representations of chemical structures, which are linked to systematic and non-systematic identifiers. It is thus crucial that different chemical identifier types represent the same compound. Inconsistencies between systematic identifiers and registered chemical structures can occur for several reasons. For example, systematic identifiers can be generated with different structure-to-identifier conversion tools, with different levels of structure standardisation, or structures and systematic identifiers can be integrated without harmonisation from different sources.

In this study we investigate the consistency of systematic identifiers of well-defined structures within and between some of the commonly used chemical resources. We also examine the effect of standardisation on this consistency.

## Methods

### Databases

For this study we selected a set of well-known publicly available small-molecule databases to cover a wide range of bioactive compounds: DrugBank [15], Chemical Entities of Biological Interest (ChEBI) [16], the Human Metabolome Database (HMDB) [17], PubChem [2], and the NCGC Pharmaceutical Collection (NPC) [18]. Table 1 shows the number of structures and corresponding systematic identifiers in each database. All data were downloaded on March 14, 2012. In this study, only compounds that had MOL files were used. Whenever available, we collected SMILES notations, InChIs strings and IUPAC names. If several SMILES notations were available for a single compound, we selected the isomeric SMILES.

**Table 1:** Number of structures (MOLs) and systematic identifier counts for databases in this study

**Database**	**MOL**	**InChI**	**SMILES**	**IUPAC**
DrugBank	6506	6391	6504	6489
ChEBI	21367	19076	19725	18798
HMDB	8534	8534	8534	7727
PubChem	5069294	5069293	5069294	4769031
NPC	8024	0	8018	0

In addition to systematic identifiers, cross-references linking records between databases were also downloaded.

The following data were extracted from the resources:

**DrugBank**[15]. The set of compounds consisted of approved drugs, experimental drugs, nutraceutical drugs, illicit drugs, and withdrawn drugs. Cross-references to other databases were extracted from the DrugCards in DrugBank.

**ChEBI**[16]. All manually checked and annotated (3 stars) structures with their corresponding systematic identifiers were downloaded. For some of these, ChEBI provides several IUPAC names. In these cases we only used the first IUPAC name in the ChEBI record for our analyses. we only used the first IUPAC name in the ChEBI record. Cross-references were obtained from the ChEBI ontology file.

**HMDB**[17]. All small-molecule metabolites with their corresponding structures were downloaded. Cross-references were extracted from the HMDB MetaboCard files.

**PubChem**[2]. Based on criteria described previously [4], a set of compounds likely to have SAR and/or other bio-annotations were downloaded from PubChem Compound. PubChem cross-references are only provided on the substance level, not on the compound level, and therefore no PubChem cross-references were used in this study.

**NPC**[18]. NPC contains the clinical approved drugs from the USA, Europe, Canada and Japan. Compounds and cross-references were downloaded through the NPC Browser 1.1.0 [18]. The export option of the NPC Browser was used to extract data in MOL and SMILES formats. NPC does not provide InChIs strings and IUPAC names.

### Consistency of systematic identifiers within a database

**Figure 1:**
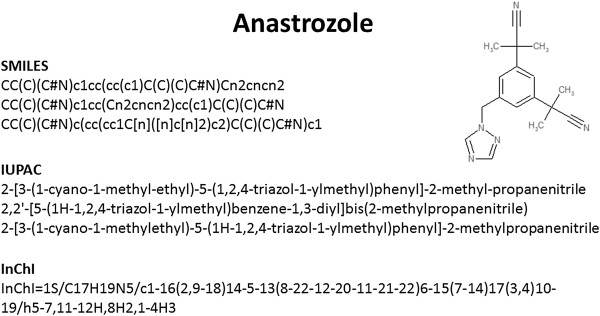
Chemical representations of anastrozole.

**Figure 2:**
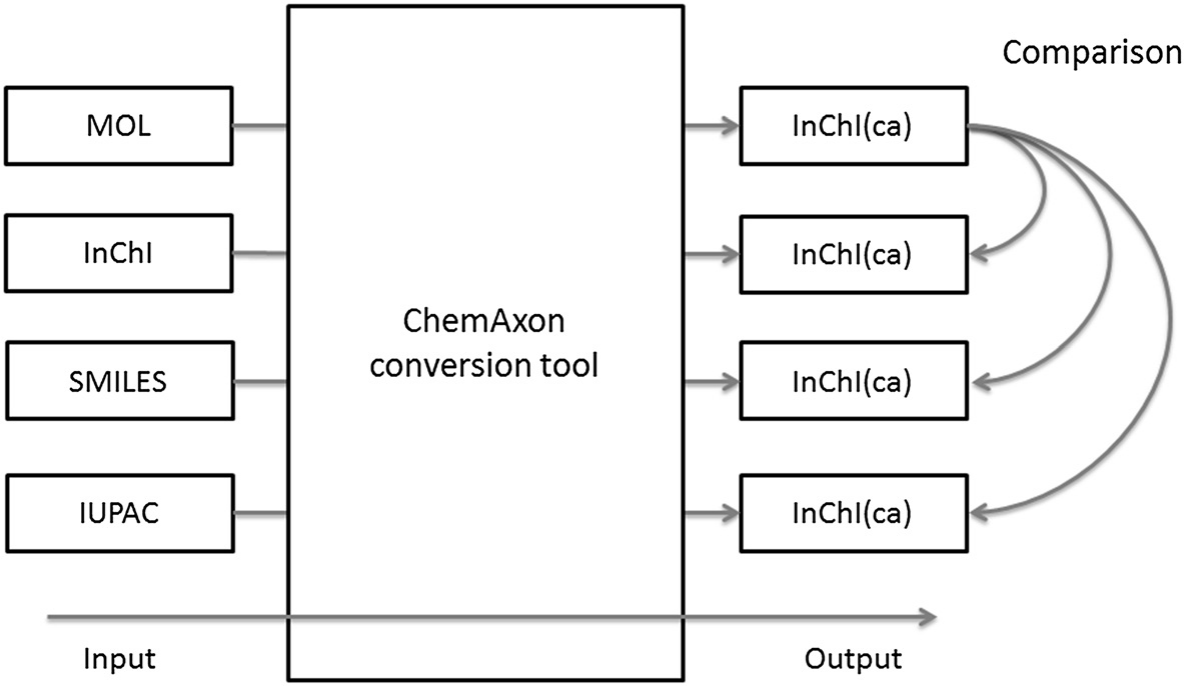
Comparison of MOL representation with systematic identifiers.

To analyse the structural representation consistency of systematic identifiers within a database, we took the MOL representation of a compound as the reference point. Ideally all associated systematic identifiers should represent the same MOL file. In this work we have used InChI strings for comparisons. InChI (International Chemical Identifier) is a structure-derived tag for a chemical compound. It is an algorithmically produced string of characters, which acts as the unique digital signature of the compound [19]. InChI software, developed by IUPAC and InChI Trust, is open-source software and the *de facto* standard for generating InChI strings [20]. This is not the case for SMILES or IUPAC names (Figure 1). Various flavours of SMILES or IUPAC names are generated by different software to represent the same molecular structure [11,21,22]. Therefore, MOL files and all systematic identifiers were converted into Standard InChIs, using InChI version 1.03, which were then used to perform all comparisons (Figure 2).

Several public and commercial cheminformatics tool-kits are currently available for structure manipulation and molecular editing [23]. We used ChemAxon’s MolConverter 5.9.1 [24], which has the necessary functionality and is freely available for academic research. For clarity, we refer to Standard InChI strings generated by ChemAxon’s MolConverter as InChI(ca).

### Consistency of systematic identifiers between databases

To analyse the consistency of systematic identifiers between databases, the cross-reference linkage of compounds was examined. Within the constraints of different chemistry business rules, the chemical entities linked together via the cross-references should represent the same structure based on their MOL representation. We compared the structures using the InChI(ca) generated from the MOLs. We did not consider cross-references where conversion to InChI(ca) failed for one or both of the MOL files. If a compound had multiple cross-references to a single database, each cross-reference was investigated independently. For cross-references to PubChem, we only considered compounds within our subset of the PubChem database.

### Standardisation

Inconsistency between systematic identifiers and their MOL representation may partly relate to the different levels of sensitivity in identifier calculation. Currently, different structure normalisation rules can be used to define compound uniqueness [25]. Unfortunately, a unified and agreed set of rules is still lacking [9]. To assess the effect of structure standardisation on the consistency of systematic identifiers within and between databases, we applied a set of rules developed by the Computer-Aided Drug Design group of the National Cancer Institute (NCI/CADD) known as FICTS rules [26,27]. These were applied to each structure and its corresponding systematic identifier.

The FICTS rules include removing small organic fragment (F), ignoring isotopic labels (I), neutralizing charges (C), generating canonical tautomers (T), or ignoring stereochemistry information (S) for a compound. If any of these rules are applied the corresponding upper-case letter is replaced with a “u” (standing for “un-sensitive” [26]). We implemented the FICTS rules using ChemAxon’s Standardizer [28]. To make the results comparable with our other analyses the rules are applied to the InChI(ca) strings.

## Results

### Conversion of systematic identifiers

Table 2 shows the percentage of successful conversion of the systematic identifiers into InChI(ca) strings by Chem-Axon’s MolConverter. This is high for MOLs, SMILES notations and InChI strings in all databases. The lower (90%) MOL conversion for ChEBI was due to the presence of query atom features such as “R” (R-groups) or “*” (= any atom). The main reason for failure in conversion of IUPAC names to Standard InChI strings was challenges for the conversion tool to handle certain structural classes such as steroids, porphyrins, and carbohydrates. The lowest value of IUPAC to InChI(ca) conversion was for HMDB.

**Table 2:** Successful conversion (in %) of MOL files and systematic identifiers to InChI(ca)

**Database**	**MOL**	**InChI**	**SMILES**	**IUPAC**
DrugBank	98.9	100	99.1	93.6
ChEBI	90.6	100	96.8	69.8
HMDB	100	99.9	100	38.1
PubChem	100	100	100	92.6
NPC	99.7	-	100	-

To investigate whether this could be improved, the same procedure was applied with another structure-to-identifier tool, the NCI Chemical Identifier Resolver [29]. This increased successful conversions slightly by 8% but still left the majority of IUPAC names in HMDB unconverted.

### Consistency of systematic identifiers within databases

For each compound in a database, we compared the InChI(ca) derived from the MOL file with the InChI(ca) strings from the corresponding systematic identifiers (Figure 2).

Table 3, shows for each database, the consistency between the MOL representation and the corresponding systematic identifiers, expressed as percentage agreement of matching InChI(ca) strings. If the InChI(ca) could not be generated for a MOL file or a systematic identifier, no comparison was done.

**Table 3:** Consistency of MOLs and systematic identifiers (in % agreement) within databases

**Database**	**MOL–InChI**	**MOL–SMILES**	**MOL–IUPAC**
DrugBank	98.2	98.5	90.0
ChEBI	96.5	96.5	75.3
HMDB	89.3	37.2	55.7
PubChem	97.7	97.8	87.2
NPC	-	93.4	-

In DrugBank there is more than 98% agreement between MOLs and their corresponding InChI strings and SMILES, while the consistency drops to around 90% for IUPAC names. PubChem and ChEBI have slightly lower agreement than DrugBank for InChI strings and SMILES notations, but the IUPAC names in ChEBI show a substantially lower agreement of 75%. The figures are lowest in HMDB with agreements of 37% for MOL-SMILES and 56% for MOL-IUPAC names. NPC only stores SMILES, which have a 93% agreement with their MOL representations.

### Standardisation

FICTS rules were applied to the InChI(ca) strings derived from the MOL files and systematic identifiers, and all comparisons were redone. Table 4 show the results. Stereochemistry has the most significant impact. For example, the consistency for MOL-SMILES notations and MOL-IUPAC names in HMDB increased with 61 and 29 percentage points. ChEBI and PubChem also show a considerable increase in agreement between IUPAC names and MOL files. In addition to stereochemistry, the changes made by standardising tautomers also improved the consistency, with the largest effect on HMDB. Charges, fragments and isotopic labels had a small or no effect on the consistency.

**Table 4:** Effect of different standardisation rules on the consistency between MOL files and systematic identifiers (in % agreement)

**Database**	**Comparison**	**FICTS**	**uICTS**	**FuCTS**	**FIuTS**	**FICuS**	**FICTu**
DrugBank	MOL–InChI	98.2	99.0	99.0	99.0	99.4	99.8
	MOL–SMILES	98.5	98.6	98.6	98.6	99.5	99.7
	MOL–IUPAC	90.0	90.1	90.0	90.1	93.5	96.2
ChEBI	MOL–InChI	96.5	98.9	98.5	98.4	99.2	99.6
	MOL–SMILES	96.5	96.6	96.6	96.6	99.6	99.8
	MOL–IUPAC	75.3	75.6	75.4	77.1	79.7	91.9
HMDB	MOL–InChI	89.3	89.8	89.7	90.3	89.9	98.5
	MOL–SMILES	37.2	37.3	37.2	38.0	43.1	98.3
	MOL–IUPAC	55.7	55.8	55.8	57.5	58.8	84.8
PubChem	MOL–InChI	97.7	97.9	97.9	97.9	99.3	99.9
	MOL–SMILES	97.8	97.9	97.9	97.8	99.2	99.9
	MOL–IUPAC	87.2	87.7	87.5	87.2	93.7	97.2
NPC	MOL–SMILES	93.4	93.5	93.4	93.4	98.0	99.8

### Consistency of systematic identifiers between databases

Table 5 shows the agreement between the MOL files for compounds with inter-database cross-references. This varies from 25.8% to 93.7%, but for most cases is around 60-75%. The low value for cross-references from NPC to PubChem can be attributed to 1527 compounds in NPC that have more than one (average 5.7, median 3) cross-reference to PubChem CIDs. The agreement for the 2475 compounds in NPC that have just one cross-reference to PubChem is 79.3%. Note that the agreement for the cross-references in DrugBank or HMDB to ChEBI is about 20% higher than the other way around.

**Table 5:** Agreement between MOL files of compounds that have a cross-reference in one database (row) to another database (column)

	**DrugBank**	**ChEBI**	**HMDB**	**PubChem**	**NPC**
DrugBank	-	72.1% (1666)	-	93.7% (4723)	-
ChEBI	54.3% (1288)	-	45.6% (114)	-	-
HMDB	-	64.0% (1433)	-	76.0% (2217)	-
PubChem	-	-	-	-	-
NPC	76.7% (1320)	-	-	25.8% (9557)	-

The number of cross-references is given in parentheses.

Since our results indicate that stereochemistry standardisation may substantially improve the consistency of systematic identifiers within databases (Table 4), we also assessed the consistency between databases after applying the FICTu rule (Table 6).

**Table 6:** Agreement between MOL files of compounds that have a cross-references in one database (row) to another database (column) after stereochemistry standardisation

	**DrugBank**	**ChEBI**	**HMDB**	**PubChem**	**NPC**
DrugBank	-	91.4%	-	95.6%	-
ChEBI	68.6%	-	93.0%	-	-
HMDB	-	82.0%	-	89.8%	-
PubChem	-	-	-	-	-
NPC	93.4%	-	-	47.6%	-

Stereochemistry annotation increases the agreement for most databases by around 15-20%. The largest increase (47.4%) is seen for cross-references linking ChEBI to HMDB.

The agreement between NPC and PubChem also increases but more than half of the cross-references still link MOL files that do not match. For compounds that have just one cross-reference the agreement increased from 79.3% to 91.0%.

## Discussion

While the importance of data quality control in chemical resources has been discussed previously [5-7,9], to our knowledge this is the first study to assess the consistency of structural representations of systematic identifiers within and between small-molecule databases. The assumption was that systematic identifiers should correspond with the registered MOL file. Standard InChI strings were used as a basis for this comparison because of the unique algorithm available, unlike for SMILES notations and IUPAC names where multiple strings can represent the same compound.

To provide comparable results and remove the influence of different structure-to-identifier software, only ChemAxon’s MolConverter [24] was used for all name conversions. Compounds where MOL files or systematic identifiers did not convert to InChI strings were disregarded. To quantify the potential influence of different structure-to-identifier software we compared the Standard InChI strings generated from the MOL files using ChemAxon’s MolConverter [24] with those of Xemistry’s CACTVS chemoinformatics toolkit [30,31]. The comparison showed 98.9% agreement for HMDB, 98.3% for PubChem, 97.6% for DrugBank, 96.4% for ChEBI, and 94.2% for NPC in cases were both tools managed to convert MOL files to InChI strings. The differences are small and likely to be caused by the way the tools handle the MOL files. We consider it unlikely that our results would essentially have changed by using another conversion tool.

The consistency of systematic identifiers with their corresponding MOL representations varies widely (Table 3). The highest agreement was obtained for DrugBank and PubChem, the lowest for HMDB. The higher consistency values for PubChem may be explained by their procedure for generating systematic identifiers [32]: starting from the MOL files, InChI strings are calculated based on the IUPAC Standard InChI software and SMILES notations and IUPAC names are generated by OpenEye software [33]. Unfortunately, because other databases do not clearly describe their procedures it remains unclear how possible differences may have affected consistency.

Application of the FICTS sensitivity rules [26] gave us further insight. We found that disregarding stereochemistry and, to a lesser extent, tautomers boosted the consistency, in particular of MOL-IUPAC names (Table 4). The other sensitivity levels had a much lower or no effect. Thus, differences in stereochemistry between MOL files and systematic identifiers appear the single most important cause of inconsistencies. For ChEBI and HMDB, the agreement between MOLs and IUPAC names remained low even with stereochemistry insensitive matching.

The consistency of systematic identifiers between databases, as measured by the agreement of MOL files in different databases linked by cross-references, ranged from 26% to 94% (Table 5). The value of cross-references lies in the consistency of the structural representation of the data and our study shows these have many errors. Disregarding stereochemistry on the registered MOL files increased the agreement, but a considerable percentage of the cross-references remained inconsistent.

Integration of different chemical databases should consider these problems. Merging databases using different structure identifiers as indexes for integration can reduce quality. Instead, a unique representation such as MOL files can be used as the basis of integration. Other systematic identifiers can be generated later on the validated structure within the database.

Inconsistencies within databases may steer curation efforts, and by combining the information on inconsistencies for a specific compound may even suggest which of the names or representations are wrong.

In a recent article by Williams et al. [9] several solutions have been proposed to reduce errors in databases. In addition to improved curation, the use of structure validation filters for incorrect valance, atom labels, aromatic bonds, charges, stereochemistry and duplication was suggested. In another recent study, O’Boyle [11] proposed a standard method to generate canonical SMILES based on InChI strings, in order to create the same canonical SMILES using different toolkits. Our results quantify the issues raised in these studies. We have shown that a set of well-defined standardisation rules is essential while constructing systematic identifiers (can gain up to 50% increase in consistency), and that stereochemistry has an important contribution to this inconsistency.

Our approach of testing the consistency of systematic identifiers is general and can be applied to other databases and may prove valuable in data curation and integration efforts. Using a similar approach, we also plan to investigate the consistency of non-systematic identifiers in chemical resources.

## Conclusions

The degree of consistency within systematic chemical identifiers varies between data sources. When building a new database, de novo recalculation is superior to recycling and creating systematic identifiers starting from the same primary structural representation (e.g. MOL) will improve the quality of the final product. Extra consideration should be taken into account if systematic identifiers are going to be used as a key index for merging databases. Well-defined and documented chemistry standardisation rules applied to all compounds can greatly decrease the number of errors and expedite integration.

Finally, we have shown that inconsistency exists between the structural representations of compounds that are linked via cross-references within databases. Inconsistency here can have deleterious effects when merging data from or cross-querying multiple databases.

## Competing interests

The authors declare that they have no competing interests.

## Authors’ contributions

SAA extracted, processed and analysed the data, and drafted the manuscript. JAK and SM supervised and coordinated the project and revised the manuscript. All authors read and approved the final manuscript.
